# A Case of Strangulated Hernia

**Published:** 1785

**Authors:** 


### II. *A Cafe of Strangulated Hernia. By Mr.* Edward Ford, *Surgeon to the Weftminfter General Difpenfary.*

R. W. of Queen Street, Golden Square, 60 years old, dropfical, and afthmatic, had been fubject to a rupture for feveral years, which he frequently found difficulty in returning. He had paid no other attention to the management of his complaint, than that of fometimes wearing a very indifferent trufs; till at length, at feven o'clock in the evening, on Tuefday the 22d of March, 1785, he found himfelf very ill, and the fymptoms were fuch as evidently pointed out a ftrangulated hernia. He complained of great ficknefs at the ftomach, with frequent inclination to vomit; he had a retention of ftools; fome degree of fever, attended with a quick and hard pulfe; and a great tenfion of the tumor. Attempts were inftantly made to replace the contents of the hernial fac within the cavity of the abdomen, but from the great pain he felt in handling of the parts, thefe efforts were foon difcontinued.

Copious bleeding, purgative medicines, adminiftered by the mouth and per anum, and warm warm fomentations to the part were tried for a few hours; but this plan procuring no alleviation of the fymptoms, recourfe was had to the warm bath, and tobacco clyfters, and at laft to the application of ice; but thefe means likewife proved ineffectual.

Early the next morning, his pulfe was become fo weak and feeble, as fcarcely to be felt; his body was covered with a cold and clammy fweat; his hiccoughs were frequent; his afpect cadaverous; and the tumor, though lefs tenfe, remained totally irreducible. About eleven o'clock, being fixteen hours from the firft fymptoms of ftrangulation, he died.

The fucceeding day his body was opened, and the fac was found to contain four inches of the jejunum highly inflamed, but not fphacelated; a large quantity of the omentum in a found ftate, and not adherent to the fac; with about half a pint of a yellowifh ferum.

The ftricture of the tendon was very tight upon the inteftine.

It will fcarcely be thought proper to draw any practical inference from the narrative of a fingle cafe; though it is obvious, from the rapid progrefs of the difeafe, that the operation for the bubonocele would have been juftifiable at any period earlier than ufual.

This cafe may ferve alfo to fhow, that the circumftance of the omentum accompanying the defcent of the inteftine, does not, in every inftance, prevent that excefs of ftrangulation to which the hernia inteftinalis is fubject; and confequently that under fuch circumftances the operation cannot, as alledged by fome of the older chirurgical writers, be poftponed with fafety.

*Golden Square,*

March 27, 1785.

