# Errata

**Published:** 1785

**Authors:** 


					p. ixoy I. Zy lor any period, read
period.?

				

